# Motor Unit Number Estimation of the Second Lumbrical Muscle in Human Hand

**DOI:** 10.3389/fphys.2022.854385

**Published:** 2022-02-22

**Authors:** Ya Zong, Zhiyuan Lu, Maoqi Chen, Lianfu Deng, Qin Xie, Ping Zhou

**Affiliations:** ^1^Department of Rehabilitation Medicine, Ruijin Hospital, Shanghai Jiao Tong University School of Medicine, Shanghai, China; ^2^Faculty of Rehabilitation Engineering, University of Health and Rehabilitation Sciences, Qingdao, China; ^3^Shanghai Key Laboratory for Prevention and Treatment of Bone and Joint Diseases, Department of Orthopaedics, Shanghai Institute of Traumatology and Orthopaedics, Ruijin Hospital, Shanghai Jiao Tong University School of Medicine, Shanghai, China

**Keywords:** compound muscle action potential, CMAP scan, motor unit number estimation, MScanFit, second lumbrical muscle

## Abstract

The number of motor units of the lumbrical muscles in human hand has not been explored. The objective of this study was to fill this gap by estimating the number of motor units in the second lumbrical muscle. Compound muscle action potential scan of the second lumbrical muscle was performed in 12 healthy subjects, with 10 of them being tested on two separate occasions. Motor unit number estimation (MUNE) was derived from the MScanFit program. The average MUNE of the second lumbrical muscle was 41.6 ± 2.1 (mean ± standard error) from 12 subjects in the first test, and 42.0 ± 2.2 from 10 of the 12 subjects in the retest, demonstrating excellent measurement reliability. Findings of the study provide novel information about the motor unit number of the second lumbrical muscle in human hand. The relatively low motor unit number in the muscle can facilitate motor unit investigations, especially at high level muscle activation.

## Introduction

The human hand lumbrical muscles include four short intrinsic muscles, attached proximally to the tendons of flexor digitorum profundus and distally to the extensor expansions (Gosling et al., 2008). The first and second lumbrical muscles (innervated by the median nerve) arise from the radial side of the most radial tendons of the flexor digitorum profundus, corresponding to the index finger and the middle finger, respectively. The third lumbrical (innervated by the ulnar nerve) arises from the ulnar side of the middle finger tendon and the radial side of the ring finger tendon, while the fourth lumbrical (innervated by the ulnar nerve) arises from ulnar side of the ring finger and the radial side of the litter finger. The function of the lumbrical muscles is to flex the metacarpophalangeal joints and extend both the proximal and distal interphalangeal joints. These actions are involved in complex hand movement, contributing to hand dexterity (Palti and Vigler, 2012). The lumbrical muscles also have important clinical relevance. For example, the difference between the median motor latency to the second lumbrical muscle and the ulnar motor latency to the interossei muscles is sensitive for diagnosis of different grades of in carpal tunnel syndrome (Brannegan and Bartt, 2007; Meena et al., 2008; Kodama et al., 2012; Ozben et al., 2012).

The number of motor units contained in the lumbrical muscles is largely unknown, despite of the fact that a range of motor unit number estimation (MUNE) methods have been developed and applied for examination of different muscles (Gooch et al., 2014; de Carvalho et al., 2018). To the best of our knowledge, no MUNE study has been performed previously for the lumbrical muscles. This study aimed to fill the gap by providing an assessment of the number of motor units in the second lumbrical muscle. Among many MUNE methods, the MScanFit MUNE was used in this study, which is based on compound muscle action potential (CMAP) scan and a model simulation of the responses (Bostock, 2016; Jacobsen et al., 2018). The MScanFit has been tested in both healthy control subjects and individuals with neuromuscular diseases, examining different muscles, including the first dorsal interosseous, abductor pollicis brevis, abductor digiti minimi, anterior tibial, abductor hallucis, and facial muscles (Li et al., 2018; Sirin et al., 2019; Habeych et al., 2020; Higashihara et al., 2020; Kristensen et al., 2020; Witt et al., 2020; Zong et al., 2021, among others). This study presents a novel application of MScanFit MUNE, and for the first time provides information about motor unit number in the lumbrical muscle, which is largely unexplored in previous literature.

## Materials and Methods

### Subjects

Twelve neurologically intact subjects (eight male and four female) without known history of neural or muscular disorders (such as carpal tunnel syndrome) participated in the study. Their mean age was 38.3 years (range: 30–66 years); mean height was 170.5 cm (range: 158–185 cm). All subjects were right-handed. The data collection was performed during the first author (YZ)‘s visiting program to University of Texas Health Science Center at Houston (UTHealth) and TIRR Memorial Hermann Hospital (Houston, TX). The protocol was approved by the Committee for Protection of Human Subjects (CPHS) at UTHealth and TIRR Memorial Hermann. All participants gave written informed consent in accordance with the Declaration of Helsinki.

### Experiment

**Figure 1:**
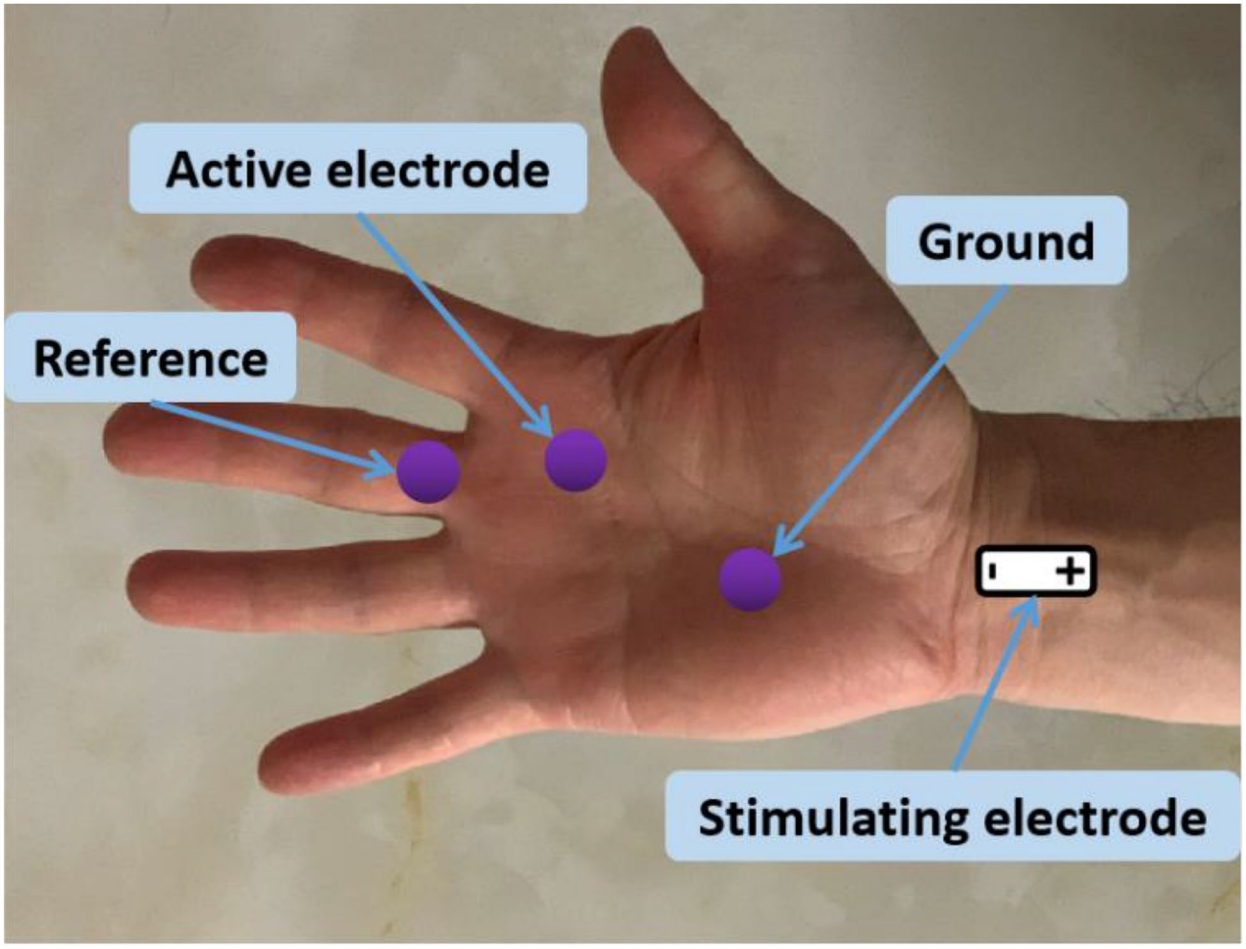
Electrode placement for CMAP scan recording of the second lumbrical muscle. Hand muscle anatomy showing the lumbricals can be found in https://learnmuscles.com/glossary/lumbricals-manus/.

The second lumbrical muscle in the dominant side of each subject was examined. During the experiment, the skin temperature maintained above 32°C. The subject was seated comfortably in a chair with shoulder and elbow flexed 90° and forearm in supination position on a height-adjustable table. The surface electrodes were placed after wipe the dead skin with alcohol pads. As illustrated in Figure 1, the active electrode was placed on the motor point of the second lumbrical muscle, and the reference electrode was placed on the surface of the proximal interphalangeal joint of the middle finger. The ground electrode was placed on the palm of hand. A standard bar electrode was placed on above the transverse carpal ligament (between the flexor carpi radialis tendon and the palmaris longus tendon) for delivering electrical stimuli to median nerve. The cathode of the electrode was positioned distally. Surgical tape and coban self-adherent wrap were used to firmly attach the bar electrode to the skin. During recording, the examined forearm was restrained in supination by Nylatex^®^ wraps (4 width) in order to minimize movement artifacts.

All the data were collected using UltraPro S100 EMG system (Natus Neurology Incorporated, Middleton, WI, United States). The built-in CMAP scan program (Synergy Research V21-1) was used to apply repetitive stimulations of the motor nerve for progressive activation of all motor units. Prior to the scan, the range of electrical stimulation intensity, i.e., S0 to S100, was appropriately adjusted to ensure coverage of the entire motor unit recruitment range. S0 corresponds to the lower end at which all-or-none response of the lowest-threshold motor unit can be recorded, while S100 corresponds to the higher end at which the maximum CMAP can be recorded. After the stimulation range was determined, the CMAP scan then started with a user-defined protocol option. In this study, the following protocol was applied for all the CMAP scans: stimulus pulse duration was 0.1 ms; stimulus number (steps) was 500, stimulus frequency was 2 Hz, and stimulus intensity declined linearly within the range. After the first CMAP scan test, all electrodes were removed. Ten of the 12 subjects were retested in a different time to determine the reliability of the CMAP scan measurements. The interval between the two tests was approximately 4–6 h (morning and afternoon on same day) for seven subjects, 1 day for one subject, and 4 days for two subjects, respectively.

### Data Analysis

The CMAP amplitudes and corresponding stimulus intensities were extracted for generating the stimulus–response curve. These data were then used by the MScanFit, which is a free software for estimating the motor unit number based on a muscle’s CMAP scan data. The software provides an interactive interface for user to change settings in MUNE calculation. First, the prescan and postscan limits were adjusted to define the beginning and ending portions of the CMAP scan curve. The next step was to determine relevant input parameters including relative spread of motor unit threshold, motor unit size limit, and number of units. All these parameters were set as default values in this study. For each CMAP scan, the program run three times with the same input model parameters but different prescan and postscan limits. The outputs of the program included derived MUNE and error score. The MUNE with the smallest percent error (required to be less than 7%) was accepted as the final estimate (Bostock, 2016).

For 10 of the 12 subjects who performed retest, the intraclass correlation coefficient (ICC) and the standard error of measurement (SEM) were calculated to assess the reliability of the CMAP and MUNE measurements. The coefficient of variation (COV) of the test and retest measurements was also computed. All the data are presented in mean ± standard error.

## Results

**Figure 2:**
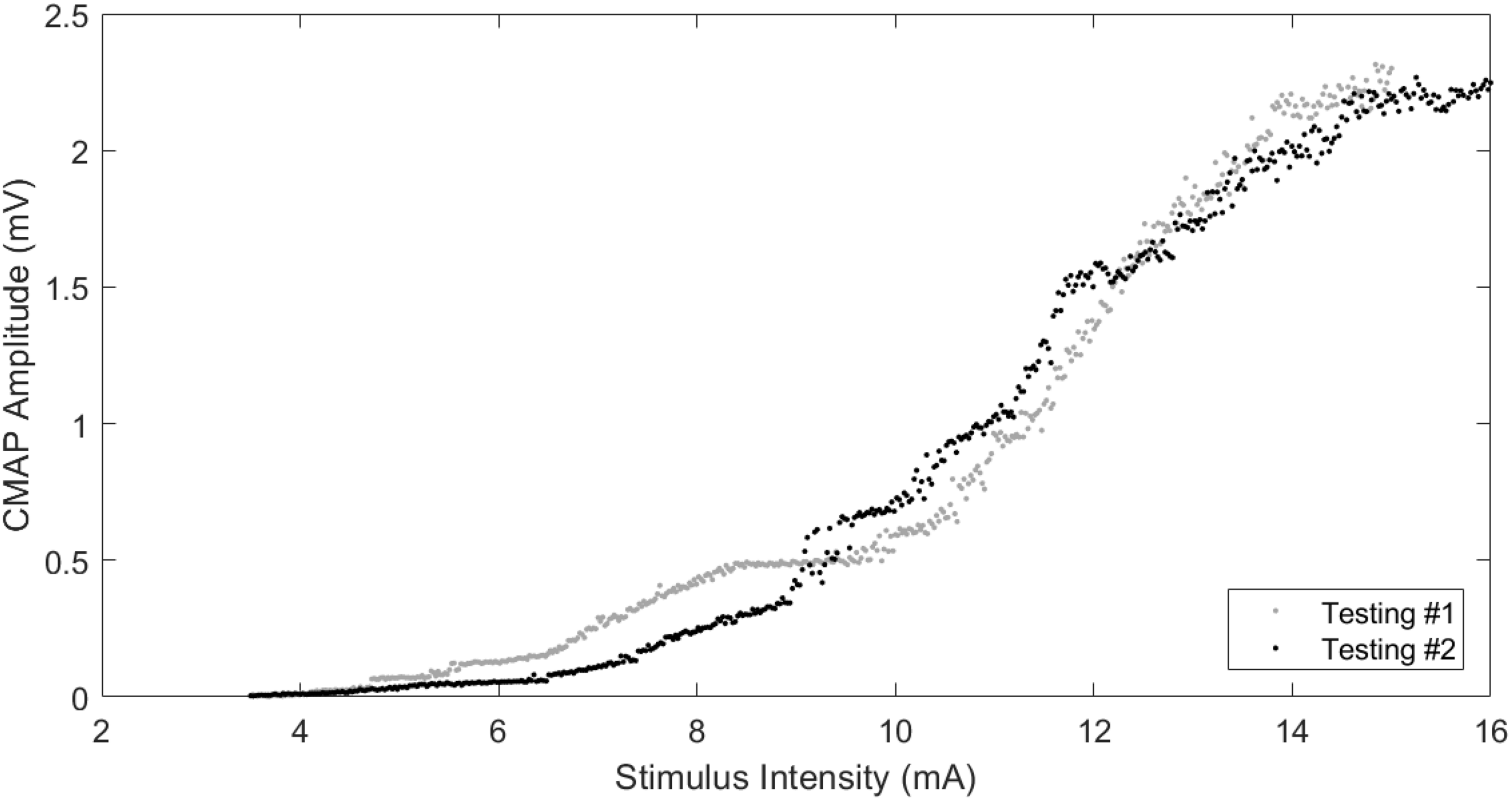
Examples of CMAP scans from the same participant on two different testings. Stimulating range: 3.5–15 mA (testing #1), 3.5–16 mA (testing #2); CMAP amplitude: 2.31 mV (testing #1), 2.27 mV (testing #2); and MUNE: 40 (testing #1), 40 (testing #2).

All participants tolerated the procedures well and completed the CMAP scans. The stimulus current for the lowest threshold (S0) and the highest threshold (S100) was 4.46 ± 0.62 mA and 14.29 ± 0.89 mA, respectively, for testing #1, and 4.10 ± 0.64 mA and 13.80 ± 1.10 mA, respectively, for testing #2 (retest). Figure 2 shows an example of the stimulus–response curves from the two tests of one participant.

**Figure 3:**
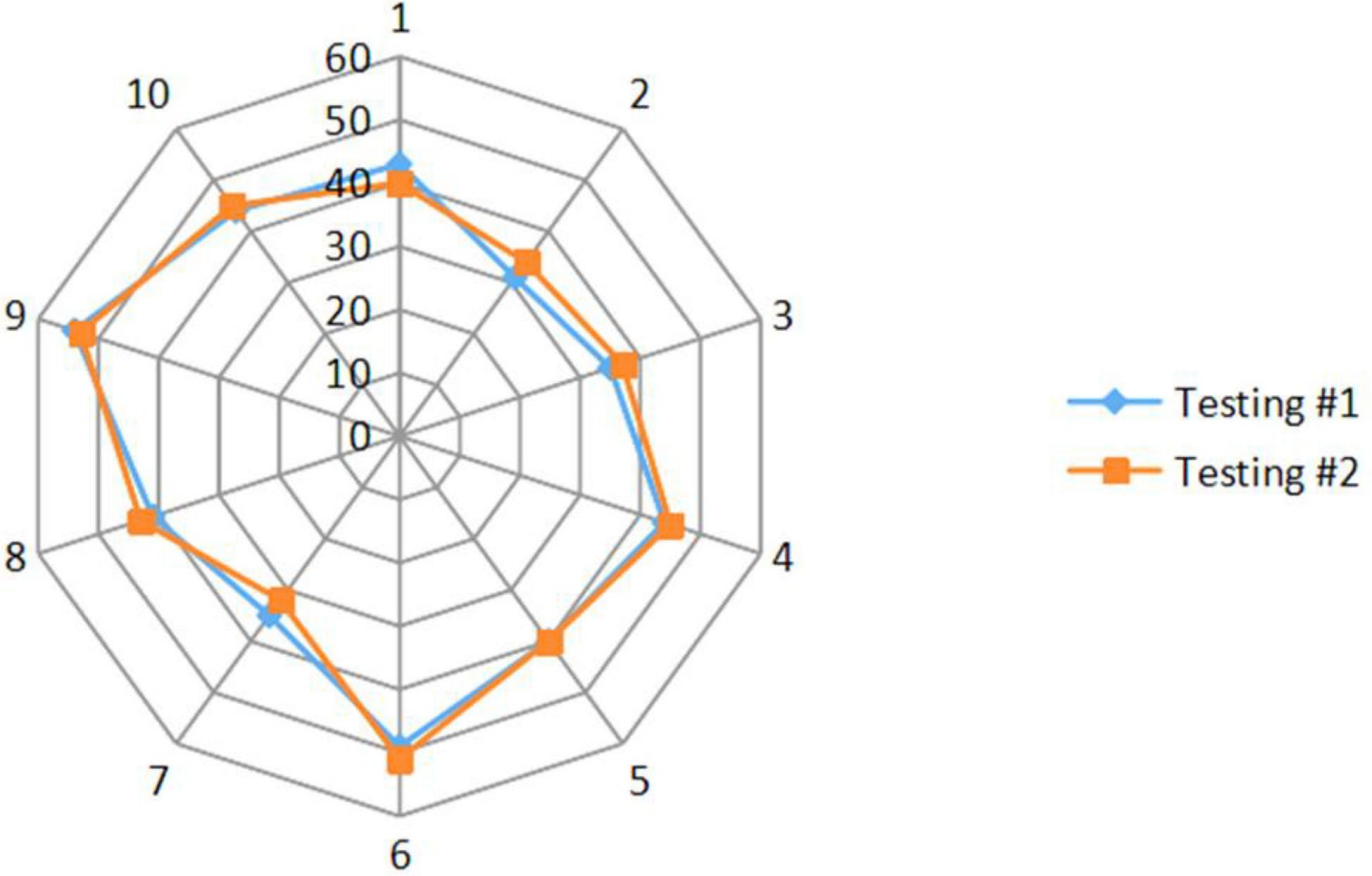
Radar plot of the MUNE distribution of the 10 subjects for both visits. The numbers around the outside of the plot represent the individual subject number (1–10), and the numbers (0–60) are on contours of equal MUNE.

The CMAP amplitude was 2.24 ± 0.19 mV (mean ± standard error) for testing #1 averaged from all the 12 subjects. For 10 of the 12 subjects participating retest, the CMAP amplitude was 2.25 ± 0.20 mV for testing #1 and 2.20 ± 0.17 mV for testing #2, respectively. The MScanFit program was applied to each CMAP scan. The derived MUNE of the second lumbrical muscle was 41.6 ± 2.1 averaged from the 12 subjects in testing #1. For 10 of the 12 subjects participating the retest, the derived MUNE was 41.6 ± 2.2 for testing #1 and 42.0 ± 2.2 for testing #2, respectively. Figure 3 shows a radar chart of the MUNE distribution of the 10 subjects for both visits. It can be observed that most of the MUNE values are within or close to 40–50, and each individual subject has similar values from the two tests. The measurement reliability of the test and retest parameters (CMAP, MUNE, largest unit size, and mean unit size) was further indicated by the ICC, SEM, and COV values, as shown in Table 1.

**Table 1:** Reliability analysis (*n* = 10, mean ± standard error used in the table).

	CMAP (mV)	MUNE	Largest unit(μV)	Mean unit(μV)
Testing #1	2.25 ± 0.20	41.6 ± 2.2	121.20 ± 14.44	51.85 ± 3.04
Testing #2	2.20 ± 0.17	42.0 ± 2.2	128.50 ± 14.75	50.40 ± 1.96
SEM	4.08%	1.05%	4.90%	1.46%
COV	(11.46 ± 2.97)%	(4.61 ± 0.94)%	(18.33 ± 3.90)%	(9.75 ± 2.73)%
ICC	0.80 (*p* = 0.002)	0.96 (*p* < 0.001)	0.82 (*p* = 0.001)	0.59 (*p* = 0.032)

*CMAP, compound muscle action potential; MUNE, motor unit number estimation; ICC, intraclass correlation coefficient; SEM, standard error of measurement; and COV, coefficient of variation*.

## Discussion

This study presents a novel assessment of the motor unit number in the second lumbrical muscle using MScanFit MUNE. In contrast to most MUNE approaches relying on mean motor unit size estimation from a small sample of motor units, MScanFit MUNE fits a model to an experimental CMAP scan curve which can provide information about full range motor unit activations of a muscle (Bostock, 2016). Using a protocol recommended in previous studies (500 steps, 2 Hz stimulus frequency, 100 ms pulse duration; Maathuis et al., 2012; Zong et al., 2020), the CMAP scan recording lasted approximately 250 s. It usually took less than 20 min to complete a CMAP scan experiment. The data processing for MScanFit could also be completed on average within several minutes. Therefore, CMAP scan and MScanFit are quick to implement once the required specific hardware and software setups are available.

The estimated number of motor units from MScanFit was 42 for the second lumbrical muscle. The test–retest analysis indicates good measurement reliability for CMAP amplitude and excellent measurement reliability for MScanFit MUNE, suggesting that MScanFit MUNE might be less dependent of CMAP amplitude, compared with traditional MUNE methods. Interestingly, we found the estimated motor unit number in this study was much lower compared with previous MUNE studies of different hand muscles, such as the first dorsal interosseous, abductor pollicis brevis, and abductor digiti minimi muscles, which have several times of the estimated motor unit number of the second lumbrical muscle (Higashihara et al., 2020; Zong et al., 2020). Compared with the interosseous muscle that has a similar function involved in fine motor control of the hand, the lumbrical is a relatively small size muscle with small cross-sectional area and weak muscle strength (Wang et al., 2014). This is reflected by the estimated motor unit number. In contrast, the first dorsal interosseous muscle was estimated to have 2 to 3 times motor unit number of the second lumbrical muscle (Zong et al., 2020).

On the other hand, it is interesting to note that the second lumbrical muscle has a similar MUNE to that of the anconeus muscle despite the much larger size of the anconeus (Stevens et al., 2014). The lumbrical muscle is involved in fine motor control of the hand. In contrast, the anconeus muscle can be viewed as an accessory extensor beside the triceps brachii, involved in posterolateral elbow stability during forearm rotation (Schneeberger et al., 2018). Therefore, compared with the lumbrical muscle, the small number of motor units in the anconeus may be more related to its function than muscle size.

The relatively low motor unit number of the muscles, such as the second lumbrical and the anconeus, may result in less complex interference pattern of EMG recording during its voluntary contractions compared with other muscles (such as biceps brachii and first dorsal interosseous muscles). This provides an attractive feature for motor unit investigation, especially at high level muscle activation. For example, it is still in debate on precise motor unit control strategies during voluntary muscle contraction (“onion-skin” vs. reverse “onion skin” phenomenon; Piotrkiewicz and Türker, 2017) primarily because it is difficult to discriminate and track single motor units at high muscle contraction levels. With relatively small number of motor units in a muscle, the second lumbrical provides a feasible model to explore such fundamental motor unit control questions using advances in EMG decomposition.

The current study is limited by only examining dominant side of a small number of young neurologically intact subjects, without assessing different sides and ages, or patients with neuromuscular disease. The test–retest analysis was also limited by different intervals and not being performed for all the subjects.

In summary, we present a MUNE study of the second lumbrical muscle in human hand of 12 healthy subjects using MScanFit based on their CMAP scan data. A relatively low MUNE (mean: 42) was found in the second lumbrical muscle. Both CMAP and MScanFit MUNE measurements of the second lumbrical muscle demonstrated high test–retest repeatability. The findings of the study provide novel information about the number of motor units in the second lumbrical muscle, which has not been explored in previous literature.

## Data Availability Statement

The original contributions presented in the study are included in the article/supplementary material, further inquiries can be directed to the corresponding authors.

## Ethics Statement

The studies involving human participants were reviewed and approved by Committee for Protection of Human Subjects (CPHS) at UTHealth and TIRR Memorial Hermann. The patients/participants provided their written informed consent to participate in this study.

## Author Contributions

YZ performed data collection, analysis, and interpretation and wrote the first draft of the manuscript. ZL, MC, and LD participated in data collection, analysis, and interpretation. QX and PZ performed study design and supervision and oversaw data collection, analysis, and interpretation. PZ revised the manuscript. All authors read, modified, and approved the final version.

## Funding

This study was supported in part by the Shanghai Municipal Key Clinical Specialty under grant number shslczdzk02701, in part by the National Nature Science Foundation of China under grant number 82102179, and in part by Shandong Provincial Natural Science Foundation under grant numbers ZR2021QH053, ZR2021QH267, and ZR2020KF012.

## Conflict of Interest

The authors declare that the research was conducted in the absence of any commercial or financial relationships that could be construed as a potential conflict of interest.

## Publisher’s Note

All claims expressed in this article are solely those of the authors and do not necessarily represent those of their affiliated organizations, or those of the publisher, the editors and the reviewers. Any product that may be evaluated in this article, or claim that may be made by its manufacturer, is not guaranteed or endorsed by the publisher.
